# Archetypal analysis of diverse *Pseudomonas aeruginosa* transcriptomes reveals adaptation in cystic fibrosis airways

**DOI:** 10.1186/1471-2105-14-279

**Published:** 2013-09-23

**Authors:** Juliane Charlotte Thøgersen, Morten Mørup, Søren Damkiær, Søren Molin, Lars Jelsbak

**Affiliations:** 1 Department of Systems Biology, Technical University of Denmark, DK-2800 Lyngby, Denmark; 2 Department of Applied Mathematics and Computer Science, Technical University of Denmark, DK-2800 Lyngby, Denmark

**Keywords:** Archetypal analysis, Gene expression, *Pseudomonas aeruginosa*, Cystic fibrosis, Hypermutators

## Abstract

**Background:**

Analysis of global gene expression by DNA microarrays is widely used in experimental molecular biology. However, the complexity of such high-dimensional data sets makes it difficult to fully understand the underlying biological features present in the data.

The aim of this study is to introduce a method for DNA microarray analysis that provides an intuitive interpretation of data through dimension reduction and pattern recognition. We present the first “Archetypal Analysis” of global gene expression. The analysis is based on microarray data from five integrated studies of *Pseudomonas aeruginosa* isolated from the airways of cystic fibrosis patients.

**Results:**

Our analysis clustered samples into distinct groups with comprehensible characteristics since the archetypes representing the individual groups are closely related to samples present in the data set. Significant changes in gene expression between different groups identified adaptive changes of the bacteria residing in the cystic fibrosis lung. The analysis suggests a similar gene expression pattern between isolates with a high mutation rate (hypermutators) despite accumulation of different mutations for these isolates. This suggests positive selection in the cystic fibrosis lung environment, and changes in gene expression for these isolates are therefore most likely related to adaptation of the bacteria.

**Conclusions:**

Archetypal analysis succeeded in identifying adaptive changes of *P. aeruginosa.* The combination of clustering and matrix factorization made it possible to reveal minor similarities among different groups of data, which other analytical methods failed to identify. We suggest that this analysis could be used to supplement current methods used to analyze DNA microarray data.

## Background

DNA microarrays simultaneously monitor expression levels of thousands of genes, and this technology is widely used in experimental molecular biology. However, the complexity of such high-dimensional data sets makes it difficult to fully comprehend the underlying biological features present in the data. Different dimension reduction techniques aim to find patterns in high complexity data sets. The choice of analytical method can influence the interpretation of the data, and it can be useful to combine different methods.

K-means clustering and principal component analysis (PCA) are techniques for unsupervised pattern recognition commonly used in microarray data analysis. K-means clustering aims to group samples (or genes) with similar behavior [1]. Each sample is then assigned to a cluster represented by a cluster centroid. PCA is an orthogonal linear transformation transforming the data into a new coordinate system where the axes are oriented to account for maximal variation in the data set. PCA decomposes data into a set of uncorrelated variables called principal components [2-4].

Clustering approaches give easy interpretable features but pay a price in terms of modeling flexibility, because each sample must be grouped in only one cluster and no intermediate between clusters is allowed. PCA on the other hand can lead to complex representations from which we learn relatively little about the data. Archetypal analysis (AA) combines the virtues of both clustering and PCA in that AA results in easy interpretable components that have added flexibility over clustering by allowing intermediates [5]. Cutler and Breiman first introduced AA in 1994, where they used AA to analyze air pollution and head shape [6]. Later, AA has been applied in the identification of extreme practices in benchmarking and market research and signal enhancement and feature extraction of IR image sequences [7,8]. Recently, AA has been shown to be useful in extracting features from different high-dimensional data sets including neuroimaging, computer vision and text mining data sets [5] and also in identifying extreme and representative human genotypes within the human population [9].

AA estimates the principle convex hull of a data set. The convex hull can be described as a minimal set of points that can wrap a given data set. The idea of AA is to find a few representative points (archetypes) in a data set such that all data can be described as a convex combination of these archetypes. The archetypes are related to experimental data but they are not necessarily observed points in the data set. Each archetype represents distinct characteristic features. Explaining data as a combination of these features can make the data set easier to interpret [5]. Unlike PCA, AA is not restricted by orthogonality, and it is possible that this method will clarify biologically meaningful features that are not discovered by PCA, while resulting in a more detailed account of the data than given by clustering approaches such as k-means clustering.

AA has been shown to be useful in extracting features from different high-dimensional data sets. So far, the method has not been applied to gene expression data despite clear advantages such as the intuitive and straightforward interpretation of the AA components. AA can be considered an unmixing approach that decomposes each observation into a weighted average of features defining distinct aspects in the data. In the related unmixing framework for gene expression data proposed in [10] the data is projected to a PCA subspace. In this subspace each observation is defined as convex combinations of features forming the simplex with smallest volume among candidate simplices that are found by an iterative boundary growing procedure that is terminated when all observations are enclosed. Contrary to this framework, AA operates directly on the full data and as the features are constrained to be convex combinations of the observations the archetypes will not in general enclose all observations.

Variation of phenotypes found in nature has recently been described as weighted averages of archetypes, where archetypes represent phenotypes that are optimized for a single adaptive task [11]. The phenotype space will often be arranged in a simple geometric shape where archetypes represent the corners, and the closer a point is to a corner the more important the corresponding task is to fitness in the organism’s habitat [11]. From this it can be concluded that it is possible to identify the tasks that are important for fitness by analyzing these corners [12]. Furthermore, the variation within a species (the combination of archetypes) reflects the different environments it inhabits [11]. The message of the paper by Shoval et al. (Science) [11] clearly illustrates the value of AA and the idea of considering a phenotype space as a combination of extreme but representative points, which is exactly the concept of this present analysis: Archetypal Analysis.

In this study, we apply AA to five gene expression data sets for *Pseudomonas aeruginosa* isolated from the lungs of cystic fibrosis patients. The five data sets were based on different experimental conditions including growth medium and growth state during cell harvesting. A method like PCA most likely captures this experimental variance in the first few components. The first components will make restrictions for the additional components due to the orthogonality constraint, and information that is linked to the real biological difference between the samples may be difficult to extract. Since AA is not restricted by orthogonality like PCA, we propose that AA will be able to extract biological information despite the different experimental conditions of the five studies. We show how AA succeeds in identifying genes that undergo changes in gene expression during evolutionary adaptation of the bacteria to the cystic fibrosis lung.

## Methods

**Figure 1:**
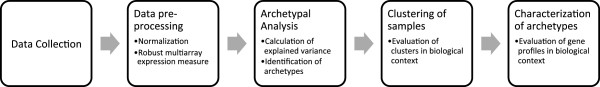
**Flow diagram of the archetypal analysis.** First, data is collected and pre-processed. Then, Archetypal Analysis is applied resulting in a clustering of samples based on the closest defined archetype. Finally, the archetypes are characterized and evaluated in a biological context.

The diagram in Figure 1 illustrates the process of AA. First data is collected and pre-processed. Pre-processing includes extraction of the raw data *cel*-files in R by use of the package *affy*[13]. Then, data is normalized using the qspline method [14] and gene expression index values are calculated using *robust multiarray average* expression measure [15]. The next stage is to apply the AA algorithm to the expression matrix and calculate explained variance in order to evaluate the solution. Once the archetypes are defined, it is possible to see how samples cluster together based on their relation to the archetypes. Finally, the archetypes can be characterized in a biological context based on their gene expression profiles. The gene expression values were not calculated relative to control strains since different control strains were used across the five analyzed studies.

### Data collection

We analyzed cDNA microarray data from four previously published *in vitro* studies (study 1–4) of *P. aeruginosa* sampled from CF lung infections. Three of the data sets were obtained from the online NCBI Gene Expression Omnibus (GEO) Database with the accession numbers GSE21966 [16], GSE31227 [17] and GSE10362 [18]. The fourth data set by Lee et al. [19] was obtained through request directly to the corresponding author. A fifth data set was generated for this study (study 5). An overview of the microarray data set is shown in Table 1.

**Table 1:** List of samples

**Sample #**	**Sample name**	**Study**	**Patient**	**Clone**	**Year**	**Mucoid**	**Mutator**	**State**^**1**^	**Medium**^**2**^	**OD**^**3**^
[1,2]	Huse_A1	Study 1	A	“A”	~1983	No	N/A	Early	SCFSM	0.4-0.5
[3,4]	Huse_A2	Study 1	A	“A”	~1984	No	N/A	Early	SCFSM	0.4-0.5
[5,6]	Huse_A3.1 (m)	Study 1	A	“A”	~1985	Yes	N/A	Early	SCFSM	0.4-0.5
[7,8]	Huse_A3.2	Study 1	A	“A”	~1985	No	N/A	Early	SCFSM	0.4-0.5
[9,10]	Huse_A4 (m)	Study 1	A	“A”	~1986	Yes	N/A	Early	SCFSM	0.4-0.5
[11,12]	Huse_B1	Study 1	B	“B”	~1983	No	N/A	Early	SCFSM	0.4-0.5
[13,14]	Huse_B2.1	Study 1	B	“B”	~1987	No	N/A	Late	SCFSM	0.4-0.5
[15,16]	Huse_B2.2	Study 1	B	“B”	~1987	No	N/A	Late	SCFSM	0.4-0.5
[17,18]	Huse_B2.3 (m)	Study 1	B	“B”	~1987	Yes	N/A	Late	SCFSM	0.4-0.5
[19,20]	Huse_B3.1 (m)	Study 1	B	“B”	~1991	Yes	N/A	Late	SCFSM	0.4-0.5
[21,22]	Huse_B3.2	Study 1	B	“B”	~1991	No	N/A	Late	SCFSM	0.4-0.5
[23,24]	Huse_B3.3 (m)	Study 1	B	“B”	~1991	Yes	N/A	Late	SCFSM	0.4-0.5
[25,26]	Huse_Ca1	Study 1	C	“Ca”	~1983	No	N/A	Early	SCFSM	0.4-0.5
[27,28]	Huse_Ca2 (m)	Study 1	C	“Ca”	~1983	Yes	N/A	Early	SCFSM	0.4-0.5
[29,30]	Huse_Cb1 (m)	Study 1	C	“Cb”	~1987	Yes	N/A	Early	SCFSM	0.4-0.5
[31,32]	Huse_Cb2	Study 1	C	“Cb”	~1987	No	N/A	Late	SCFSM	0.4-0.5
[33,34]	Huse_Cb3 (m)	Study 1	C	“Cb”	~1987	Yes	N/A	Late	SCFSM	0.4-0.5
[35-36]	Huse_PA14	Study 1	N/A	“PA14”	N/A	No	N/A	wt	SCFSM	0.4-0.5
[37-38]	Huse_PAO1	Study 1	N/A	“PAO1”	N/A	No	N/A	wt	SCFSM	0.4-0.5
[39-41]	Yang_PAO1	Study 2	N/A	“PAO1”	N/A	No	N/A	wt	LB	0.5
[42-47]	Yang_CF510-2006	Study 2	N/A	“WTB”	N/A	No	N/A	N/A	LB	0.5
[48-50]	Yang_B6.0	Study 2	B6	“B6”	~2005	No	N/A	Early	LB	0.5
[51-53]	Yang_B6.4	Study 2	B6	“B6”	~2007	No	N/A	Early	LB	0.5
[54-56]	Yang_B12.0	Study 2	B12	“B12”	~2005	No	N/A	Early	LB	0.5
[57-59]	Yang_B12.4	Study 2	B12	“B12”	~2007	No	N/A	Early	LB	0.5
[60-62]	Yang_B12.7	Study 2	B12	“B12”	~2009	No	N/A	Early	LB	0.5
[63-65]	Yang_B38.1	Study 2	B38	“B38”	~2005	No	N/A	Early	LB	0.5
[66-68]	Yang_B38.2 (m)	Study 2	B38	“B38”	N/A	Yes	N/A	Early	LB	0.5
[69-71]	Yang_B38.2	Study 2	B38	“B38”	~2005	No	N/A	Early	LB	0.5
[72-74]	Yang_B38.6 (m)	Study 2	B38	“B38”	~2006	Yes	N/A	Early	LB	0.5
[75-77]	Yang_CF43-1973	Study 2	CF43	“DK2”	1973	No	N/A	Early	LB	0.5
[78-80]	Yang_CF66-1973	Study 2	CF66	“DK2”	1973	No	N/A	Late	LB	0.5
[81-83]	Yang_CF105_1973	Study 2	CF105	“DK2”	1973	No	N/A	Early	LB	0.5
[84-86]	Yang_CF114_1973	Study 2	CF114	“DK2”	1973	No	N/A	Early	LB	0.5
[87-89]	Yang_CF30-1979	Study 2	CF30	“DK2”	1979	No	N/A	Late	LB	0.5
[90-92]	Yang_CF173-1984	Study 2	CF173	”DK2”	1984	No	N/A	Late	LB	0.5
[93-95]	Yang_CF333-1991	Study 2	CF333	”DK2”	1991	No	N/A	Late	LB	0.5
[96-98]	Yang_CF66-1992	Study 2	CF66	“DK2”	1992	No	N/A	Late	LB	0.5
[99-101]	Yang_CF333_1997	Study 2	CF333	“DK2”	1997	No	N/A	Late	LB	0.5
[102-104]	Yang_CF173-2002	Study 2	CF173	“DK2”	2002	No	N/A	Late	LB	0.5
[105-107]	Yang_CF243-2002	Study 2	CF243	“DK2”	2002	No	N/A	Late	LB	0.5
[108-110]	Yang_CF333-2003	Study 2	CF333	“DK2”	2003	No	N/A	Late	LB	0.5
[111-113]	Yang_CF173-2005	Study 2	CF173	“DK2”	2005	No	N/A	Late	LB	0.5
[114-116]	Yang_CF333-2005	Study 2	CF333	“DK2”	2005	No	N/A	Late	LB	0.5
[117-119]	Yang_CF333-2007.1	Study 2	CF333	“DK2”	2007	No	N/A	Late	LB	0.5
[120-122]	Yang_CF333-2007.2 (m)	Study 2	CF333	“DK2”	2007	Yes	N/A	Late	LB	0.5
[123-125]	Yang_CF333-2007.3 (m)	Study 2	CF333	“DK2”	2007	Yes	N/A	Late	LB	0.5
[126-128]	Yang_CF66-2008	Study 2	CF66	“DK2”	2008	No	N/A	Late	LB	0.5
[129-131]	SD_CF211-1997 (m)	Study 5	CF211	“DK2”	1997	Yes	N/A	Late	LB	1
[132-134]	SD_CF211-1997	Study 5	CF211	“DK2”	1997	No	N/A	Late	LB	1
[135-137]	SD_CF211-2006 (m)	Study 5	CF211	“DK2”	2006	Yes	N/A	Late	LB	1
[138-140]	SD_CF211-2006	Study 5	CF211	“DK2”	2006	No	N/A	Late	LB	1
[141-142]	Lee_CF30-1992 (m)	Study 3	CF30	“DK1”	1973	Yes	No	Late	BB	1
[143-144]	Lee_CF30-1992	Study 3	CF30	“DK1”	1973	No	No	Late	BB	1
[145-146]	Lee_CF30-2001 (m)	Study 3	CF30	“DK1”	2001	Yes	No	Late	BB	1
[147-148]	Lee_CF30-2001	Study 3	CF30	“DK1”	2001	No	No	Late	BB	1
[149-150]	Lee_CF46-1988 (m)	Study 3	CF46	“DK1”	1988	Yes	No	Late	BB	1
[151-152]	Lee_CF46-1988	Study 3	CF46	“DK1”	1988	No	No	Late	BB	1
[153-154]	Lee_CF46-1997 (m) HYP	Study 3	CF46	“DK1”	1997	Yes	Yes	Late	BB	1
[155-156]	Lee_CF46-1997 HYP	Study 3	CF46	“DK1”	1997	No	Yes	Late	BB	1
[157-158]	Lee_CF128-1992 (m)	Study 3	CF128	“DK1”	1992	Yes	No	Late	BB	1
[159-160]	Lee_CF128-1992 HYP	Study 3	CF128	“DK1”	1992	No	Yes	Late	BB	1
[161-162]	Lee_CF128-2002 (m)	Study 3	CF128	“DK1”	2002	Yes	No	Late	BB	1
[163-164]	Lee_CF128-2002 HYP	Study 3	CF128	“DK1”	2002	No	Yes	Late	BB	1
[165-167]	Hob_1998	Study 4	M	“M”	1998	No	No	Late	LB	>3
[168–170]	Hob_1998 (m)	Study 4	M	“M”	1998	Yes	No	Late	LB	>3
[171-173]	Hob_1999	Study 4	M	“M”	1999	No	No	Late	LB	>3
[174–176]	Hob_2001	Study 4	M	“M”	2001	No	No	Late	LB	>3
[177-179]	Hob_1999 HYP	Study 4	M	“M”	1999	No	Yes	Late	LB	>3
[180-182]	Hob_2001.1 HYP	Study 4	M	“M”	2001	No	Yes	Late	LB	>3
[183-185]	Hob_2001.2 HYP	Study 4	M	“M”	2001	No	Yes	Late	LB	>3
[186-188]	Hob_2001.3 HYP	Study 4	M	“M”	2001	No	Yes	Late	LB	>3

^1^Adaptation state (early or late).

^2^Growth medium for the experiments: Synthetic Cystic Fibrosis Sputum Medium (SCFSM), Luria-Bertani Broth (LB), or Beef Broth (BB).

^3^Optical density (OD) at 600 nm during cell harvest.

Study 1 [16]*.* This gene expression data set consists of 17 samples (in duplicates) representing clonal isolates sampled from three CF patients on timescales ranging from 3 months to 8 years. Two of the patients each harbored a unique clone (A and B), whereas a strain replacement occurred in the third patient, and two individual clones Ca and Cb were therefore isolated from this patient. For each isolate, information about colony morphology is available, and for the present analysis, we grouped these morphotypes into two categories: Mucoid ('mucoid’ morphotypes) and non-mucoid ('dwarf’ and 'classic’ morphotypes).

The experimental procedures are fully described by Huse et al. [16]. In brief, cells were grown in synthetic cystic fibrosis sputum medium (SCFSM) to an optical density read at 600 nm (OD_600_) of 0.4-0.5 prior to Affymetrix *P. aeruginosa* GeneChip microarray analysis. The strains *P. aeruginosa* PAO1 and *P. aeruginosa* PA14 (referred to as PAO1 and PA14 respectively) were included as controls in their study. PAO1 was originally isolated from a burn wound [20] and has been widely used as a reference strain for studies of *P. aeruginosa.* PA14 is a highly virulent laboratory strain that most likely represents an environmental strain of *P. aeruginosa*, although it has also been isolated from CF lungs in Europe [21,22]*.*

Study 2 [17]*.* This data set consists of different clonal lineages isolated from the lungs of CF patients (B6, B12, B38, CF30, CF46, CF66, CF105, CF114, CF173, CF211, CF243, CF333 and CF506) between 1973 and 2008 spanning early stage infection to chronic stage infection [23]. Many of the isolates from study 2 share the same clonal type called “DK2”. The data set consists of 29 samples in triplicates. One group of samples was isolated from CF children between 2006 and 2008 and these isolates represent early stage infection. Each isolate was characterized based on two colony morphotypes; mucoid and non-mucoid. The data set includes a sequential mucoid and non-mucoid paired strain, where the non-mucoid strain (B38-2NM) was generated *in vitro* by allelic replacement of its *mucA* allele [24]. Cells were grown in Luria-Bertani (LB) medium to OD_600_ of 0.5 (OD_600_ = 1 for samples #129-140) prior to Affymetrix *P. aeruginosa* GeneChip microarray analysis. PAO1 was included as control in this study.

Study 3 [19]*.* This data set consists of twelve clonally related, sequential mucoid and non-mucoid paired *P. aeruginosa* isolates. The isolates were obtained from three CF patients. All isolates from study 3 share the same clonal type called “DK1”. Cells were grown in beef broth (BB) to an OD_600_ of 1 prior to Affymetrix *P. aeruginosa* GeneChip microarray analysis. Each experiment was done in duplicate. Isolates with high mutation rates (hereafter, “hypermutators”) were identified within the data set.

Study 4 [18]*.* This data set consists of eight sequential isogenic isolates recovered over a period of three to five years from a single CF patient (patient M). The isolates included both hypermutators and non-hypermutators and one isolate was mucoid. Cells were grown in LB medium and harvested during late-logarithmic growth phase at optical density above 3. Each sample was triplicated.

Study 5 (this study). This data set consists of four isolates from the same patient (CF211). The isolates are two mucoid/non-mucoid pairs isolated together in 1997 and 2006 respectively. Cells were grown in BB to an OD_600_ of 1 prior to Affymetrix *P. aeruginosa* GeneChip microarray analysis. Microarray data were generated using Affymetrix protocols as previously described [23]. Each experiment was done in triplicates. The isolates share the same clone type “DK2” as many of the isolates from study 2, but the experimental conditions are similar to those in study 3.

### Archetypal analysis

The fundamental principle of AA is briefly introduced below. AA is fully described by Cutler and Breiman [6]. AA is defined by the decomposition

$$\begin{array}{c} X\approx \mathrm{XCS},\\ s.t.\;C\;\underline{>}\;0,\sum_{n=1}^{N}c_{\mathrm{nd}}=1,\;S\;\underline{>}\;0,\sum_{d=1}^{D}S_{\mathrm{dn}}=1. \end{array}$$

Where we use the notation ***S*** *≥* ***0*** to denote that the entries of a matrix ***S*** are constrained non-negative. The archetypes (components) are given as the columns of the matrix ***A*** defined by ***A*** *=* ***XC*** such that the columns of **A** are formed by convex combinations of the samples.

A *K-*component AA finds a matrix with elements *A*_*mk*_ defining *K M*-dimensional archetypes and each data point can be represented by a convex combination of these archetypes. Each archetype thereby has a specific gene profile that is saved in the *k*’th column of ***A***, i.e. ***a***_*k*_. The coefficients (α_1_, α_2_, .., α_*K*_) for a given data point ***x***_*n*_ are saved in the nth column of the matrix ***S***, i.e. ***s***_*n*_, with elements *S*_*kn*_. The values of these coefficients range from 0 to 1 and the sum of the coefficients equals 1.

The AA algorithm as for PCA and k-means attempts to minimize the residual sum of squares (RSS).

$$\mathrm{RSS}=\sum_{m=1,n=1}^{M,N}{\left(X‒\mathrm{AS}\right)}_{\mathrm{mn}}^{2}={║X‒\mathrm{AS}║}_{F}^{2}$$

Where M is the number of attributes and N the number of observations.

Determining the characteristics of each of the archetypes can clarify the features of the data set.

### Principal component analysis and k-means clustering

Principal component analysis and k-means clustering were applied to the same data set.

Principal component analysis is given by the decomposition

$$\begin{array}{c} X\approx \mathrm{AS},\\ \begin{array}{cc} s.t.\;A^{T}A=I, & SS^{T}=D, \end{array} \end{array}$$

where ***I*** is the identity matrix and ***D*** is a diagonal matrix with the elements in the diagonal are sorted according to their magnitude.

In k-means clustering ***S*** is constrained to be a binary assignment matrix such that ***A*** = ***XS***^***T***^ (***SS***^***T***^)^-1^ represents the Euclidean centers of each cluster.

### Number of components

**Figure 2:**
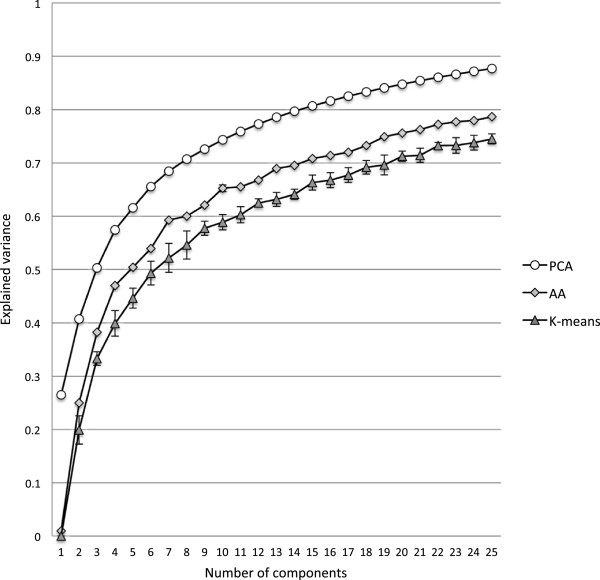
**Explained variance.** The explained variance plotted as a function of number of components for principal component analysis (PCA) archetypal analysis (AA) and k-means clustering (K-means). The plotted values are the mean of 10 repeated iterations. The standard deviations are indicated with error bars for k-means clustering. The standard deviations for archetypal analysis are very small and therefore not visible.

For AA, it is necessary to set the number of components prior to analysis similar to k-means clustering. Our choice of archetype component was guided by plotting the explained variance as a function of number of components (Figure 2). For the purpose of this study, we chose to analyze seven components, which kept the number of components at a minimum while at the same time explaining a large part (59.3%) of the variance. The standard deviation between 10 repeated iterations is very low, which suggests that the solution is robust. The explained variance for PCA and k-means clustering with seven components were 54.4% and 68.4% respectively. As expected, the PCA model, which is the most flexible of the considered models, has a higher explained variance than AA that in turn has a higher explained variance than the more restricted k-means clustering.

As a quality measure the deviation between the n^th^ original data point ***x***_*n*_ and the derived data point ***XCs***_*n*_ based on the seven archetypes was calculated. The measure is given as the Explained Sample Variance $\left(\mathrm{ESV}=\cdot \frac{{║x_{n}║}_{F}^{2}-║x_{n}-XCs_{n}║{}_{F}^{2}))}{{║x_{n}║}_{F}^{2}}\cdot \right)$ ranging between 0 and 1 where 1 is a perfect match. By evaluating these *ESV* values*,* it is possible to state which data points are well described by the model. No conclusions should be made for data points where *ESV* is low, because these data points are poorly described by the model.

### Characterization of archetypes

Each archetype was characterized based on its specific gene profile. This was done by identifying genes with statistically significant transcriptional changes. Genes with more than a two-fold change in expression value, compared to the mean expression of the respective gene for all samples, were indicated as up-regulated whereas genes with less than 0.5-fold change were indicated as down-regulated. Genes were assigned to 26 different gene ontology (GO) classes based on the gene annotation for *P. aeruginosa* PAO1 from the Pseudomonas Genome Database [25]. If a gene was assigned to more than one GO class it was re-assigned to the most overall GO class (Additional file 1: Table S1). GO classes that were over-represented within the group of up-or down-regulated genes were identified by the Hypergeometric distribution test with significance level p = 0.01 [26].

### Matlab code

The methods mentioned above were implemented in Matlab unless otherwise stated. The Matlab Code for AA is available online at http://www.mortenmorup.dk. This code estimates ***C*** and ***S*** using a projected gradient descent iterative approach initialized by the FurthestSum procedure, for details see also [5]. A brief description of the Matlab Code is listed in Additional file 2.

## Results and discussion

To explore the value of Archetypal Analysis in gene expression studies, we assembled microarray data from five separate studies of clinical *P. aeruginosa* sampled from CF lung infections [16-19]. These studies measured global gene expression of different clonal *P. aeruginosa* isolates under diverse *in vitro* growth conditions. The studied bacterial isolates exhibited different clinically relevant phenotypes such as mucoidy and hypermutability, were different clone types, and were isolated from patients at different time points in relation to disease progression (Table 1).

### Defining archetypes in the data set

**Figure 3:**
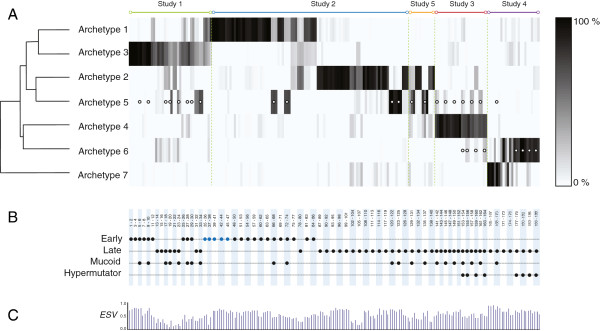
**Heatmap of archetypal analysis results. A**. The relation of each sample to the seven different archetypes shown as a heat map of the coefficient matrix ***S***. Each row represents one of the archetypes and each column represents a sample. The corresponding studies are listed above the heat map. The shading indicates how much the individual archetypes contribute to each sample. A strong correlation close to 100% is black whereas a low or no correlation is white. The white dots that appear in archetype 5 indicate mucoid samples and the white dots that appear in archetype 6 indicate samples that are hypermutators. **B**. Phenotypic data i.e. adaptation state (early/late), mucoid/non-mucoid and hypermutability are indicated. Reference strains (PAO1 and PA14 from study 1 and 2) are categorized as “Early”, but they are distinguished with a blue color. **C**. The values of Explained Sample Variance (*ESV*) are included to show how well the samples are described by the model.

AA was performed on a data set with 188 samples in total (sum of duplicates and triplicates) using the code provided in [5] and additional codes that are available online (see Methods section). Seven archetypes were identified for the integrated data set. The contribution of the individual archetypes to each sample is visualized as a heat map of the coefficient matrix ***S*** in Figure 3. The relation between the gene profiles of the seven archetypes is shown in a dendrogram based on hierarchical clustering (Figure 3).

### Archetypal analysis separates study 2 into two groups representing adapted and non-adapted isolates respectively

Archetype 1, 2 and 5 represent samples from study 2. This appears from Figure 3 by these samples having coefficients close to one (100%) in one of the three archetypes. Study 2 is composed of samples that were isolated from cystic fibrosis patients from the Danish CF clinic between 1973 and 2008 [17]. The samples from this study can be divided into two groups; one group representing isolates from early infection (hereafter referred to as 'non-adapted’ isolates), and one group representing isolates from long-term chronic infection (hereafter referred to as 'adapted’ isolates). Archetype 1 represents non-adapted isolates from study 2 including the reference strain PAO1 and an isolate called CF510-2006 that is considered as an ancestor to many of the isolates from study 2 [27]. CF510-2006 has phenotypic characteristics similar to wild type environmental *P. aeruginosa* strains [27]. Archetype 2 represents adapted isolates from study 2. One of the isolates from study 2 (triplicate samples #78-80) is best explained by archetype 2, although it was isolated in 1973 and is considered as an early isolate with respect to time of isolation. However, from genomic studies of study 2 it is known that this isolate has two mutations located in the genes *rpoN* and *mucA* and these mutations are common to the adapted isolates and they are associated with an adapted phenotype [23]. This isolate therefore can justifiably be considered to belong to the group of adapted isolates. The archetypes 1 and 2 thereby successfully cluster study 2 into two distinct groups based on adaptation level.

Some of the samples from both groups of study 2 are also, to a greater or lesser degree, based on archetype 5. These samples all have a mucoid phenotype characterized by an over-production of alginate. The transition from a non-mucoid to a mucoid phenotype is often observed during adaptation of the bacteria to the CF lung and this shift is important for establishment of chronic infections [28].

### Characterization of archetype 1, 2 and 5

**Figure 4:**
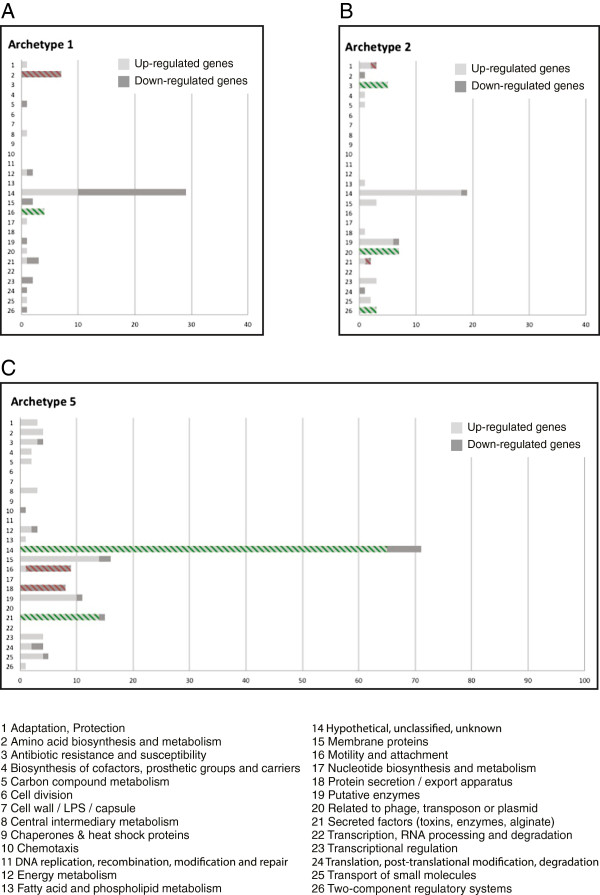
**Characterization of archetype 1, 2 and 5.** Number of up-and down-regulated genes within 26 gene ontology classes for archetype 1 **(A)**, archetype 2 **(B)** and archetype 5 **(C)**. Enriched gene-ontology classes are cross-hatched in green and red for up-and down-regulated genes respectively. The values on the x-axes are number of genes.

We next studied the up-and down-regulated genes within each archetype to find patterns that would suggest specific biological properties associated with archetype 1, 2 and 5. Figure 4 shows the distribution of significantly up- and down-regulated genes with respect to GO classes for these three archetypes. GO classes that were over-represented within the group of up-or down-regulated genes were identified by Hypergeometric distribution test [26].

A full list of up-and down-regulated genes for all archetypes can be found in Additional file 1: Table S1. From the archetype characterization in Figure 4A, it appears that the early strains represented by archetype 1 have a high expression of genes belonging to the GO class “Motility and Attachment”. At the same time, they have a low expression of genes related to “Amino acid biosynthesis and metabolism”. The adapted strains represented by archetype 2 are characterized by up-regulation of genes related to “Antibiotic resistance and susceptibility”, “Two-component regulatory systems” and genes “Related to phage, transposon and plasmid” (Figure 4B). Down-regulated genes belong to the functional classes “Adaptation, Protection” and “Secreted factors”. These observations are in agreement with earlier studies examining the phenotypic differences between non-adapted and adapted isolates [17,23,24,29]. Archetype 5 was primarily characterized by a strong up-regulation of genes related to alginate biosynthesis belonging to the GO class “Secreted factors” (Figure 4C). This is in agreement with the mucoid phenotype, characterized by overproduction of alginate that is observed for all the samples that have an apparent coefficient for this archetype. This archetype is also characterized by up-regulation of many genes encoding hypothetical proteins and down-regulation of genes involved in “Motility and Attachment” and “Protein secretion”.

AA succeeds in clustering study 2 into biologically meaningful groups. At the same time, it is easy to extract biological features important for all groups. So far, the AA analysis is verified since the characteristics of the archetypes 1, 2 and 5 are consistent with results from genotypic and phenotypic studies of study 2 [23].

The identification of these genes thereby validates this model and we are able to find biological characteristics of the different samples by analyzing the archetypes. For each of the archetypes the lists of up-and down-regulated genes also include genes encoding hypothetical proteins and it is possible that such genes are also involved in the adaptation process. For archetype 5 there are a large proportion of up-regulated genes belonging to the GO class “hypothetical proteins”. Further experimental studies are required to understand the function of these genes and their relation to the adaptation process.

### Parallel adaptation processes are observed between study 1 and study 2

**Figure 5:**
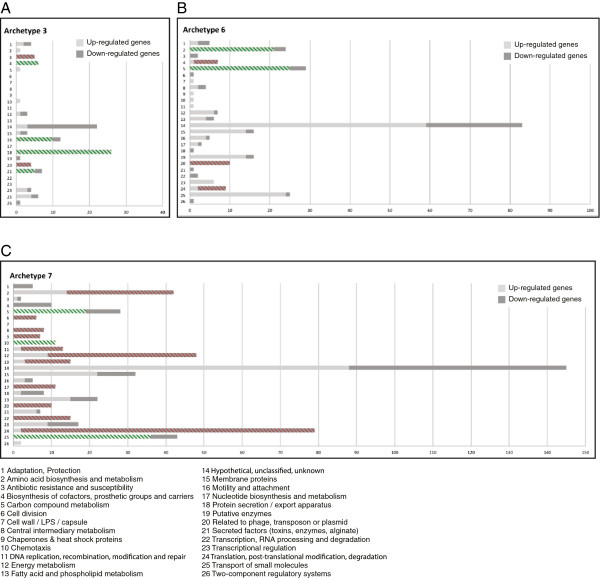
**Characterization of archetype 3, 6 and 7.** Number of up-and down-regulated genes within 26 gene ontology classes for each archetype for archetype 3 **(A)**, archetype 6 **(B)** and archetype 7 **(C)**. Enriched gene-ontology classes are cross-hatched in green and red for up-and down-regulated genes respectively. The values on the x-axes are number of genes.

Archetype 3 is defined close to a subset of samples (#1-10) from study 1. These samples all have the same genotype (clone A) and they are considered as non-adapted since they were isolated early during the infection history of the patient (cf. Table 1) [16]. This archetype is characterized by up-regulation of genes belonging to the GO classes “Motility and attachment”, “Protein secretion” and “Secreted factors” and many of these genes are related to type III secretion and pilin biosynthesis. Archetype 3 is characterized by down-regulation of “Antibiotic resistance and susceptibility” and genes “Related to phage, transposon and plasmid” (Figure 5A).

Archetype 1 and 3 represent early isolates from study 2 and study 1 respectively. The two archetypes share characteristics with respect to up-regulation of “Motility and attachment” and down-regulation of genes with relation to “Adaptation and antibiotic resistance”. Hierarchical clustering of the seven archetypes also groups archetype 1 and 3 together shown in a dendrogram in Figure 3A.

Samples #11-15 are the earliest isolates of another clone (clone B) from study 1 and they are also closely related to archetype 3. Samples #25-30 represent early isolates of two other clones (clone Ca and Cb) from study 1 and they are best described by archetype 3. However, they also show recognizable coefficients (weak bands in Figure 3A) for archetype 1, which also indicates the similarity between the non-adapted samples from study 1 and study 2. This also applies to samples #9-10 that are the latest isolate of the clonal group A from study 1. The reference strains PA14 and PAO1 are included in study 1 and they are best described by archetype 1 that also represents PAO1 samples from study 2. Differences between data from study 1 and study 2 are therefore most likely to be due to different clonal lineages more than experimental differences. Samples #17-24 are late isolates of clone B from study 1. Unfortunately, the samples are poorly described by the model, as indicated by the low *ESV* values. However, the samples show similarity to archetype 2 representing the adapted isolates from study 2. Together these findings suggest that the adaptation processes from the two studies 1 and 2 are parallel.

Samples #5-6, #9-10, and #27-30 are reported as mucoid but they do not appear to be similar to the mucoid isolates from study 2, where archetype 5 identified all the mucoid isolates. However, archetype 5 succeeds in identifying the mucoid isolates for samples #20, #23-24 (weak indication) and #33-34.

### Archetypal analysis groups the samples from study 5 together with its clonal relatives from study 2 despite different experimental conditions

Archetype 2 and 5 best describe study 5. The samples that have a coefficient close to one for archetype 5 are mucoid and this is consistent to the results for study 2. The non-mucoid isolates are close to archetype 2, which represents non-mucoid isolates from study 2 sharing the same clonal type as the isolates in study 5. This shows a strong consistency between study 2 and 5, although study 5 was performed under experimental conditions similar to those in study 3.

The five analyzed studies were performed under diverse experimental conditions including different media types. We compare the characteristics of the seven described archetypes and some of the differences are most likely due to the effect of the different media. This study does not account for how the different media alone affect the transcriptome. However, when we compare the different archetypes we have seen that the samples cluster more into groups of clonally related bacteria than into clusters of samples exposed to the same experimental procedure e.g. PAO1 in study 1 and study 2 and the samples from study 2 and study 5. The effect of the diverse media types does therefore not override the real biological relation between the bacteria and we justify comparing the samples from the five studies despite different experimental procedures. Future investigations of clonally related bacteria may further examine the effect from the media alone on the transcriptome.

### A single archetype represents hypermutators for study 3 and study 4

Archetype 4 mainly represents study 3. Study 3 is also composed of samples derived from the Danish CF clinic representing adapted isolates as for study 2. However, the samples share another clonal type (DK1) and the experiments are performed under different conditions than those used for study 2. The differences between archetype 2 and 4 are most likely due to clonal differences more than experimental differences since the same differences in experimental conditions did not separate study 2 and study 5 in this analysis. A plot of enriched gene ontology classes for archetype 4 similar to plots in Figure 4 is accessible in Additional file 3. The samples from study 3 differ from each other as some of them have a minor recognizable coefficient in archetype 5 or archetype 6. Archetype 5 represented the mucoid isolates from study 2. All the samples from study 3 that have a recognizable coefficient for archetype 5 are in fact mucoid. In this case, knowledge from one study can be transferred to another study despite the different experimental conditions and clonal types between the two studies. Archetype 6 represents samples from study 4. The samples that are closest to this archetype are all hypermutators.

The samples from study 3 with recognizable coefficients for archetype 6 are also hypermutators. One of the hypermutator samples in study 3 is not identified as having a recognizable coefficient for archetype 6. However, this sample stands out from the rest of the hypermutators by also being mucoid. The analysis thereby suggests an archetype that is able to characterize hypermutators in general. The similarity of the hypermutators could be due to similar selective pressures present in the lung environments of CF patients. This analysis could suggest that the hypermutators follow the same path of evolution despite many changes arising as a consequence of mutations.

Hypermutation is often due to mutations in the *mutS* or *mutL* genes that are part of the mismatch repair system [30]. The hypermutator trait is often observed for adapted strains of *P. aeruginosa*[18,19,31,32] and the high mutation rate is thought to be advantageous in the changing host environment due to acceleration of adaptation [18,30]. A reason for the hypermutators to develop a similar adaptive phenotype, but different from the adapted non-mutators, could be the chance of obtaining a combination of multiple adaptive mutations at one time, which is less likely for strains with a normal mutation rate [32]. Another possibility is that the *mutS* gene or the *mutL* gene possesses a regulatory function that is altered due to the mutation in the respective gene. There is some evidence that bacteria can sense the missing mismatch repair function and this will influence transcriptional regulation [33]. This would make a fingerprint on the gene expression profiles for the hypermutators resulting in similarity between the gene expression profiles. A third possibility is that the mutation targets of the hypermutators are biased due to for example a preference of specific transversions and transitions and other phenomena [34]. This analysis suggests that there is a common phenotypic trait between the hypermutators. The underlying reason needs further investigation.

### Amino acid biosynthesis and metabolism are important for adaptation to the cystic fibrosis lung

The characteristics of archetype 6 might be used to better understand the features shared by the hypermutators. However, the experimental procedure used for study 4 is markedly changed since the samples are harvested in late-logarithmic growth phase (optical density read at 600 nm ≥ 3) compared with exponential growth conditions for study 1, 2, 3 and 5. The observed up-and down-regulated genes can therefore be ascribed to changes during the transition from exponential to stationary growth phase more than to changes due to accumulated mutations. In order to exclude effects due to the growth phase, archetype 6 is compared to archetype 7. Both archetypes represent samples from study 4. Archetype 7 is mainly represented by non-hypermutators that constitute isogenic pairs to the samples represented by archetype 6.

For archetype 7 many GO classes are overrepresented by either up-or downregulated genes (Figure 5C). This is most likely due to the different growth conditions in study 4 compared to the other four studies. The profile of archetype 7 is very different from the other studies suggesting significant changes in the transcriptome due to the change in growth conditions. If we consider archetype 6 (Figure 5B), we do not observe the same dramatic changes. This can also be seen from the dendrogram in Figure 3 where archetype 6 is closer to the remaining archetypes than archetype 7. This could indicate that the hypermutators represented by archetype 6 are not that sensitive to the changes in growth conditions compared to the non-mutators, represented by archetype 7. An explanation for this could be that the hypermutators possess mutations in regulatory genes that make the gene expression less sensitive to the surrounding conditions, in this case growth phase and cell density.

Archetype 6 is also characterized by up-regulation of genes involved in the GO-class 2 ('Amino acid biosynthesis and metabolism’) and down-regulation of genes from GO-class 4 ('Biosynthesis of cofactors, prosthetic groups and carriers’) suggesting that these are important during adaptation of *P. aeruginosa* to the CF lung for the hypermutators.

These findings are to a certain extent similar to what Hoboth et al. [18] found when comparing hypermutator isolates with a non-mutator isolate. They also found amino acid biosynthesis and metabolism to be important for adaptation together with other metabolic pathways [18]. One difference in the comparison is that they compared the transcriptomic profiles directly, whereas in this analysis the two proposed archetypal gene expression profiles for archetype 6 and 7 are compared, where archetype 6 represents hypermutators and archetype 7 represents non-mutators. We suggest that the characteristics of the archetype 6 are representative for general hypermutator characteristics, since archetype 6 accounts for hypermutators across different clonal types and across different experimental conditions (study 3 and 4). The gene expression profile of archetype 6 therefore most likely can be linked to the hypermutator trait and its influence on adaptation in the CF lung.

### Archetypal analysis supplements principal component analysis and k-means clustering

**Figure 6:**
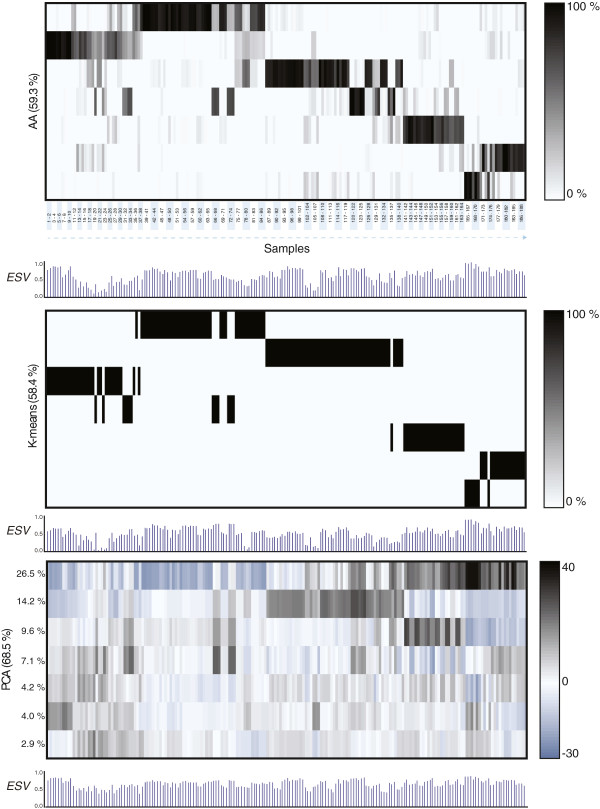
**Comparison between archetypal analysis, principal component analysis and k-means clustering.** Visual representation of a seven-component analysis using archetypal analysis (AA), principal component analysis (PCA) and k-means clustering (K-means). Explained sample variance (*ESV*) for each analysis is included. For each PCA component the contribution to explained variance is indicated. The explained variance for a seven component analysis is indicated in brackets for each analysis.

Results of k-means clustering and PCA of the data set are illustrated together with the results of AA in Figure 6. The results of k-means clustering show how samples are divided into seven groups. The clustering pattern is similar to the pattern from AA but each sample is assigned to only one cluster making k-means clustering rigid compared to AA.

**Figure 7:**
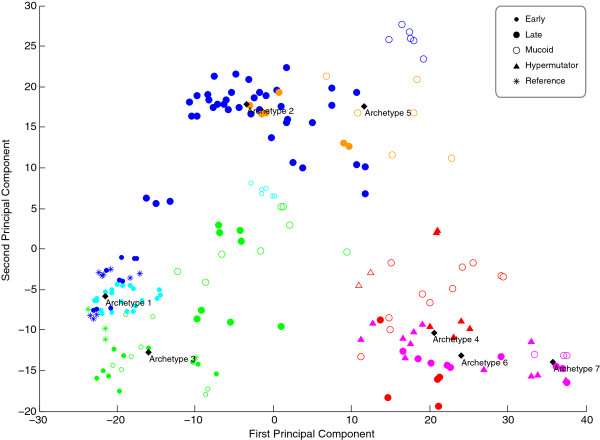
**Principal component analysis scatter plot.** Each sample is plotted with respect to the loadings of first and second PCA component. The seven archetypes from archetypal analysis are transformed into the PCA space through a basis transformation. Each Study is indicated with a specific color. Study 1: GREEN, study 2: CYAN (samples #48-74) and BLUE (samples #75-128), study 3: RED, study 4: MAGENTA, study 5: ORANGE. The phenotypes are indicated with symbols as “Early”, “Late”, “Mucoid” and “Hypermutator”. The reference strains PAO1 and PA14 from study 1 and 2 are indicated with a symbol as “Reference”.

PCA captures most of the explained variance in the first three components (50.3%). However, the components do not give an apparent grouping of samples in Figure 6. PCA solutions are often visualized by plotting the first two components in a two-dimensional scatter plot as shown in Figure 7. Together the first two components account for 40.7% of the variance present in the data set. From the scatter plot it is neither possible to see any grouping correlated to the mucoid phenotype nor the hypermutator phenotype as identified by AA. This illustrates the value of AA compared to PCA. For the present analysis we were fortunate to know some phenotypic traits (mucoidy and hypermutability) of the samples in the data set. These properties were captured by AA. Even if this information was not available it would still be possible to suggest similarities within the data set based on AA. A drawback of AA and k-means compared to PCA is that the choice of the number of components influences how the components are defined while the iterative estimation procedures for extracting the components may terminate at suboptimal solutions. As the archetypes are constrained to be convex combinations of the observations AA relies on the presence of observations that well represent the distinct aspects in the data.

## Conclusions

This is the first time Archetypal Analysis has been applied to analysis of gene expression data. Seven archetypes were able to extract the main characteristics of the dataset. The results show that Archetypal Analysis is successful in clustering of data into biologically meaningful groups. At the same time, the analysis is strengthened by matrix factorization making it possible to describe data points as a combination of archetypes.

Archetype 1 and 2 represent non-adapted and adapted isolates respectively, and characterization of the two archetypes identifies the main changes during adaptation of the bacteria to the CF lung. In this study, it is shown that one archetype represents a group of hypermutators (result of clustering) and other data points share characteristics with this group (result of factorization) enabling identification of hypermutators from another group. The analysis provides results that are easy to interpret and we suggest that this analysis could be used to supplement current methods of gene expression analysis.

## Availability of supporting data

The Matlab code for our method is freely available online on the website http://www.mortenmorup.dk.

## Competing interests

The authors declared that they have no competing interests.

## Authors’ contributions

JCT participated in the design of the study, performed the statistical analysis and wrote the manuscript. MM participated in the statistical analysis and helped to draft the manuscript. SD designed and performed DNA microarray experiments. LJ and SM participated in the design of the study and helped to draft the manuscript. All authors read and approved the final manuscript.

## Supplementary Material

Additional file 1: Table S1 List of up-and down-regulated genes for each archetype. Click here for file

Additional file 2 Short description of Matlab scripts. Click here for file

Additional file 3 Enriched gene ontology classes for archetype 4. Click here for file
