# Supplementary material for: Archetypal analysis of diverse Pseudomonas aeruginosa transcriptomes reveals adaptation in cystic fibrosis airways
Source: BMC Bioinformatics. 2013 Sep 23;14:279. doi: 10.1186/1471-2105-14-279 (PMC3870984; doi:10.1186/1471-2105-14-279)
Supplement: Additional file 2 — Short description of Matlab scripts. [file 1471-2105-14-279-S2.docx]

**Additional file 2:** Short description of Matlab scripts.

*GeneArc.m*

The script *GeneArc.m* imports microarray data from a specified text file. This text file contains the expression matrix with the gene expression values for all samples. The number of components for the analysis should be given as input before. Archetypal analysis of the data set is executed. The *A* and *S* matrices are computed using the function PCHA and the explained variance for the analysis is calculated. PCHA was described by Mørup and Hansen (2011), and Matlab code is available. The matrix *S* is illustrated as a heat map, which allows easy detection of sample clusters. Results from Principal component analysis (PCA) and K-means clustering are also displayed as heat maps for comparison.

*Varexp.m*

This script is useful for determination of number of components. *Varexp.m* calculates the explained variance for a 1 to *k* component archetypal analysis and displays explained variance as a function of number of components. The maximal number of components, *k,* must be given as input. The results are displayed as an average of a specified number of runs. Similar plots for PCA and k-means clustering are made. For k-means clustering a high value of *k* can result in empty clusters, which will interrupt the calculations. In this case, a different value of *k* can be defined for k-means clustering and the calculations can proceed.

*GeneList.m*

The script *GeneList.m* finds significantly up- and down-regulated genes for each archetype compared to the mean values of all samples. The archetypes must be defined using the script *GeneArc.m* before this script is applied*.* Genes that are found significantly up-regulated are saved in a matrix "Genes_high" whereas genes that are significantly down-regulated are saved in a matrix "Genes_low". In addition to this, information for each gene can be extracted from a specified data file including gene annotation, pathways and functional classes belonging to each gene. This information is saved in two matrices called "Genes_high_list" and "Genes_low_list" for up- and down-regulated genes respectively. The log2 expression values of these genes are listed in two matrices called "Genes_value_high" and "Genes_value_low".
