# Supplementary material for: Archetypal analysis of diverse Pseudomonas aeruginosa transcriptomes reveals adaptation in cystic fibrosis airways
Source: BMC Bioinformatics. 2013 Sep 23;14:279. doi: 10.1186/1471-2105-14-279 (PMC3870984; doi:10.1186/1471-2105-14-279)
Supplement: Additional file 3 — Enriched gene ontology classes for archetype 4. [file 1471-2105-14-279-S3.docx]

**Additional file 3:** Enriched gene ontology classes for archetype 4

| 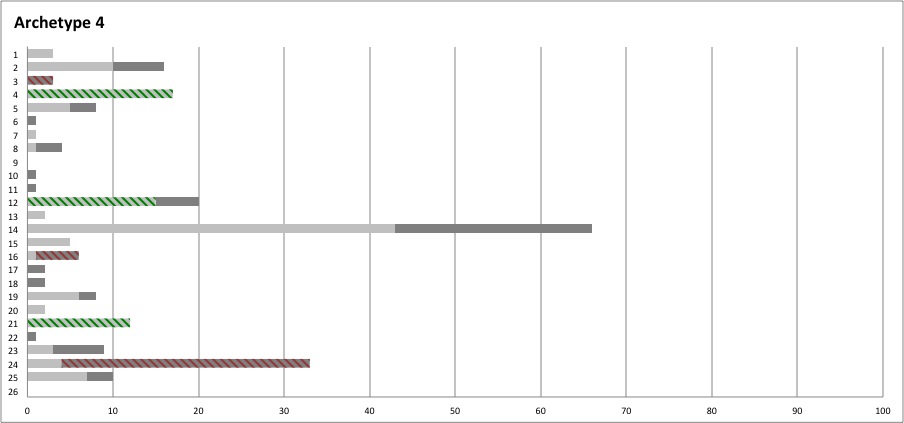 | |
| --- | --- |
| 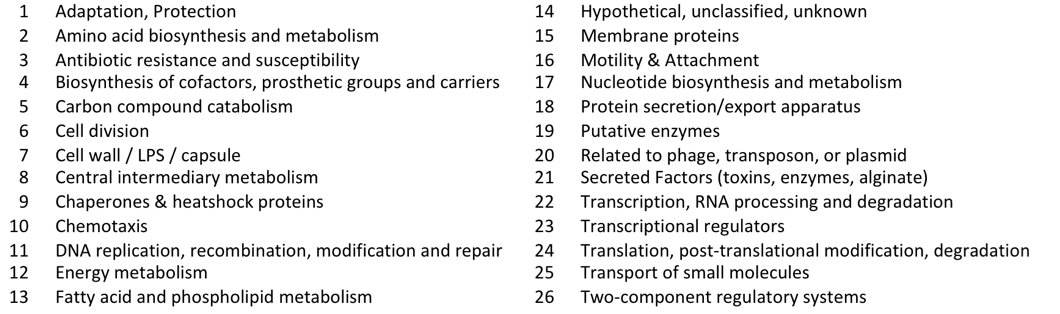 | 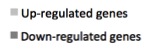 |

**Figure S3-1. Characterization of Archetype 4.**

Number of up- and down-regulated genes within 26 gene ontology classes for archetype 4. Enriched gene-ontology classes are high-lighted in green and red for up- and down-regulated genes respectively.
